# Effects of Noise and Vibration Due to the Hokuriku Shinkansen Railway on the Living Environment: A Socio-Acoustic Survey One Year after the Opening

**DOI:** 10.3390/ijerph18157794

**Published:** 2021-07-22

**Authors:** Takashi Morihara, Shigenori Yokoshima, Yasunao Matsumoto

**Affiliations:** 1 Department of Architecture, National Institute of Technology, Ishikawa College, Ishikawa 929-0392, Japan; 2 Kanagawa Environmental Research Center, Kanagawa 254-0014, Japan; yokoshima.7c7q@pref.kanagawa.lg.jp; 3 Department of Civil and Environmental Engineering, Saitama University, Saitama 338-8570, Japan; ymatsu@mail.saitama-u.ac.jp

**Keywords:** high-speed train, noise, ground vibration, social survey, annoyance, exposure–response relationship, logistic regression analysis

## Abstract

One year after the opening of the Hokuriku Shinkansen (high-speed) railway, in 2016, we conducted a social survey targeting the residents of detached houses along the rail. Noise and vibration exposure levels were estimated at outdoor points closest to the noise source side of the houses. Of the 1980 people contacted, there were 1022 valid respondents. The purpose of this research was to investigate the relationship between noise and vibration exposure and community responses. The results demonstrated that the noise annoyance and daily activity disturbances of residents living in areas without a conventional railway are higher than those of residents living in areas running parallel to a conventional railway line. This tendency was remarkable, especially for areas with high vibration exposure caused by the Shinkansen railway. There was no difference between before and after the opening of the Shinkansen railway in the evaluation of housing satisfaction, or regarding the preference for the residential area and quietness around the house. However, since the survey before the opening was conducted only in the Ishikawa site, it will be necessary to conduct before-and-after surveys in areas where there are no conventional railways, and where the speed of the Shinkansen is fast.

## 1. Introduction

Since the opening of the Tokaido Shinkansen Line in 1964, the Shinkansen railway system has significantly increased its transportation capacity, and the Shinkansen line network has continuously expanded in Japan. Hokuriku Shinkansen is one of the new Shinkansen lines. The Hokuriku Shinkansen high-speed railway began operating between Tokyo and Kanazawa, Japan, in March 2015. The railway runs a 12-car set rolling stock and exhibits a maximum speed of 260 km/h. There are plans to extend the Hokuriku Shinkansen line to Shin-Osaka. Although the Shinkansen railway is a convenient mode of transportation, the noise and vibrations caused by passing trains can disturb residents living along the railway line. Environmental quality standards for the Shinkansen super-express railway noise have been reported since 1975 [1]; the standards are 70 dB or lower for the peak noise level in areas mainly used for residential purposes and 75 dB or lower in other areas. A vibration guideline of 70 dB was recommended by the Environment Agency in 1976. These environmental quality standards and recommendations provide standard and guideline values, respectively. The evaluation index for general noise environmental quality standards is *L*_Aeq_, and *L*_den_ is used for aircraft noise. Thus, the indicators used in Japan differ according to the noise source type. Therefore, those in which noise and vibration environments have been comprehensively assessed are necessary.

Many social surveys have been conducted on community responses to Shinkansen railway noise and vibrations [2,3,4,5,6,7]. Tamura [2] indicated that Shinkansen railways were evaluated more negatively than conventional railways in areas where railway noise was a major factor in the sound environment. Yokoshima et al. [3] reported that the community response to Shinkansen railway noise was stronger than the response to other types of ground transportation noise. Yano et al. [4] conducted a social survey and measured the noise and vibrations along the Sanyo Shinkansen line, demonstrating that the Shinkansen railway emitted greater ground vibrations than conventional lines at the same noise level. Most surveys were performed after the Shinkansen network began operations, and only one study compared the residential environment before and after the opening of the Kyushu Shinkansen railway, an older section of the Shinkansen railway network [5,6]. They sought to capture community responses to the change in the elevated conventional railway running parallel to the Kyushu Shinkansen. Yokoshima et al. [7] reanalyzed the data of six social surveys conducted in Japan from 1995 to 2013 on the Shinkansen railway and confirmed the combined effect of noise and vibration exposure on the annoyance level. They reported that noise annoyance in the low-vibration group was lower than that in the high-vibration group. Considering the combined effect of noise and vibration on community response, Öhrström et al. [8] and Gidlöf-Gunnarsson et al. [9] reported the results of social surveys along the Swedish railway. Both studies demonstrated that the noise annoyance level was higher for residents in areas with vibrations than for residents in areas without railway vibrations. Peris et al. [10] also conducted a social survey on railways in the United Kingdom, demonstrating that the percentage of highly annoyed residents for *L*_den_ was higher in areas with larger vibration dose values. Experimental studies have also been conducted on the combined effect of noise and vibration exposure (e.g., Howarth et al. [11], Paulsen et al. [12], Lee et al. [13], Maigrot et al. [14], and Morihara et al. [15]). These studies reported that simultaneous exposure to both noise and vibrations results in a higher annoyance level and activity disturbance than exposure to noise or vibration alone, depending on the conditions. In other words, it may be necessary to include the effects of vibration from transportation when establishing an environmental quality standard value for noise.

Owing to the development of transportation networks, many residential areas are affected not only by single transportation noise but by various factors such as vibrations and air pollution from multiple transportation modes. Morihara et al. [16] investigated the living environment before the opening of the Hokuriku Shinkansen line in Ishikawa, Japan. Conventional railways currently operate in areas where the Shinkansen is scheduled to open. The results revealed that more residents were satisfied with their living environment (37%) than dissatisfied (16%). Moreover, 61% of respondents positively evaluated their residential environment. Few studies have examined the effects of noise and vibration on a living environment before and after the opening of a high-speed railway.

The purpose of this study is to clarify the following two points. The first is to demonstrate noise exposure–response relationships on noise annoyance and activity disturbances after the opening of the Shinkansen railway. This study is a report of the survey results one year afterwards, and we will attempt to capture the changes in the community responses of residents by conducting further continuous surveys in the future. The second is to verify whether the evaluations of housing satisfaction, preference for a residential area, and quietness around the house changed before and after the opening. The limitations of this study are the inclusion of community responses of the area where the Shinkansen runs slowly in the analyzed data, as well as the fact that all the surveyed houses were detached houses; therefore, the present results are due to the residents.

This paper has been divided into five parts, including this introductory section. The second section is concerned with the materials and methodology used for this study. The third section presents relationships between noise exposure and community response in the living environment one year after the opening of the Shinkansen railway. In particular, we focused on the effects of vibration due to the Shinkansen railway and the existence of a conventional railway. In addition, the results of examining the effects of noise and vibration on the housing satisfaction, preference for a residential area, and quietness around the house are also shown. The fourth section discusses the results. Finally, the conclusion provides a summary and critique of the findings.

## 2. Methods

### 2.1. Social Survey

**Figure 1:**
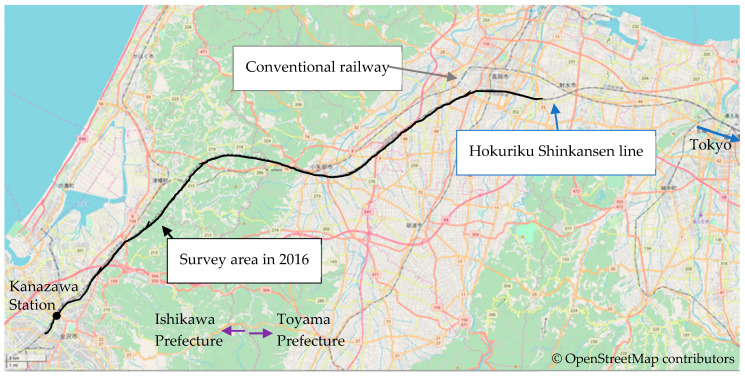
Route of Hokuriku Shinkansen and area of social survey.

We conducted the social survey in November 2016. The survey sites were located in a residential area along the Hokuriku Shinkansen railway in the Ishikawa and Toyama prefectures in northern Japan (Figure 1). The Ishikawa site corresponded to the 2007 survey sites [16], and a conventional railway line runs near many of the surveyed houses. Conversely, the newly surveyed Toyama site is a quiet residential area, and a conventional railway does not run near the site. The Shinkansen high-speed railway line is elevated above ground level at both sites. At that time, 210 conventional railways passed per day, and the maximum speed was 110 km/h (about 60 km/h on average). All conventional railways ran on the ground, and this study did not estimate the noise and vibration exposure levels.

The targeted houses were all detached houses within 150 m of the Hokuriku Shinkansen railway. If there were no houses within 150 m, we targeted the first row of houses up to 210 m away from the railway line. Respondents were selected from commercial residential maps, and one person per household was selected using the nearest birthday principle. The questionnaire consisted of 43 questions, was distributed by mail, and was titled the “Living Environment Survey.” The questions addressed the housing, residential environment, environmental pollution, daily activity disturbance, lifestyle, and demographic variables. The questions regarding noise and vibration used a five-point verbal scale (“not at all”, “slightly”, “moderately”, “very”, and “extremely”), following the International Organization for Standardization technical specification 15,666 [17,18]. In this survey, to investigate the effects of self-reported sleep disturbance due to the passage of the Hokuriku Shinkansen railway, we enquired about disturbances related to falling asleep, awakening, and awakening frequency. A five-point verbal scale similar to that for noise annoyance was used to evaluate these disturbances, except for the awakening frequency, which was asked in five intervals (1: not at all; 2: 2–3 times a year; 3: 2–3 times a month; 4: 2–3 times a week; 5: almost every day).

### 2.2. Estimations of Noise and Vibration Exposures

The Hokuriku Shinkansen railway has a maximum speed of 260 km/h, and the survey area has an elevated railway structure. Noise and vibration measurements were obtained to estimate the noise and vibration exposures of the target houses. The noise exposure levels, i.e., the maximum A-weighted and S-weighted sound pressure level (*L*_ASmax_), were estimated for the target houses using the prediction method developed by Nagakura et al. [19], and the predicted values were adjusted according to the values measured at 25 m from the center line of the proximity track.

Vibration levels, defined by the Japanese industrial standard using a reference acceleration of 10^−5^ m/s^2^, were estimated using the distance attenuation predictions that were calculated using the obtained measured values [20]. In other words, this was 20 dB different from ISO based on 10^−6^ m/s^2^. The maximum vibration level in the vertical direction (*L*_Vzmax_) on the ground surface was measured at nearly the same points as the noise measurements. The maximum index (*L*_Vmax_) was calculated as the mean value of the top 50% of the measured *L*_Vzmax_ values. The field surveys were conducted in May 2017 at representative locations in each area, which were then classified into 11 areas along the route in the Ishikawa and Toyama prefectures, considering the structure height and train speed. In each area, measurement points were provided from 12.5 m to 100 m, with reference to the close orbit center, sound level meters (for example, NL-31, 32, 42, and 62, RION, Tokyo, Japan) and vibration level meters (VM-53 and VM-55, RION, Tokyo, Japan), which were used at each measurement point. Both exposure levels encompassed the outdoor levels of the target housing closest to the rail track.

**Figure 2:**
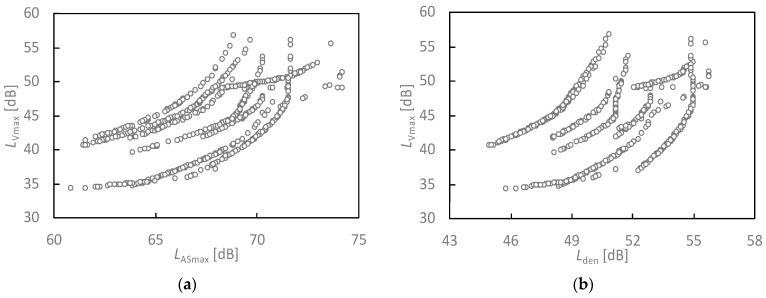
Relationships between noise exposure level and vibration level. (**a**) Relationship between *L*_ASmax_ and *L*_Vmax_; (**b**) Relationship between *L*_den_ and *L*_Vmax_.

Figure 2 displays the relationship between noise and vibration level due to the Hokuriku Shinkansen railway of each house obtained by the above evaluations. The *X*-axis is the mean of the maximum noise level measured for the slow dynamic characteristic (left, day–evening–night-equivalent sound level), and the *Y*-axis is the ground vibration level in the vertical direction. The noise level range was 60–75 dB *L*_ASmax_, 44–55 dB *L*_den_, and 34–44 dB *L*_night_ in the survey area. The range of the vibration level was from 34 dB to 57 dB. There were 114 houses with vibration levels above 50 dB, and 209 houses with noise levels above 70 dB.

### 2.3. Exposure–Community Response Relationships and Statistical Analysis

A cross tabulation was performed to examine the relationship between noise levels and high noise annoyance, self-reported sleep disturbances, and other activity disturbances. This study defined the upper 28% of the annoyance scale as highly annoyed (HA), and the 72% HA cutoff was calculated by using randomly selected responses to the second category from the top of the five-point verbal scale [21]. Highly disturbed was also defined using the same method as that of HA. Multiple logistic regression analysis was used to verify the significance of parameters such as noise levels, vibration levels, and the existence of conventional lines to annoyance and activity disturbances using IBM SPSS Statics 25. Each model incorporated the noise level, vibration level, the interaction between noise level and vibration level, and the interaction between noise level and the existence of conventional lines. These models also included age, gender, family size, and noise sensitivity (WNS-6B [22]) as adjusted variables. This study dealt with sleep disturbance as the maximum response of the falling asleep and awakening variables. High sleep disturbance (HSD) was defined in the same way as %HA.

In addition, the questions in the social survey included evaluations of housing satisfaction, preference for the residential area, and quietness surrounding the house. In this study, these three items were considered to be related to noise annoyance. These three items were assessed using question words and evaluation scales, as illustrated in Table 1. In the multiple regression analysis, the categories were divided. Using data from the same respondents as in the 2007 survey [16], we will investigate whether the evaluation of these negative sides changes after the opening of the Hokuriku Shinkansen railway, using cross-tabulation and Fisher’s exact test.

## 3. Results

### 3.1. Demographic Data and Exposure Levels

A total of 1022 people responded to the questionnaires, with a response rate of 51.6%. The respondents were predominantly male (56%), which was the same result as that of the previous survey [16], and 90% of the respondents were over 40 years old (Table 2). These results reflect the dominant demographics of people living in detached houses in regional towns and cities in Japan. We used the WNS-6B scale [22] to assess sensitivity to noise. A cutoff point of 4/5 on the WNS-6B scale was used. Of these data, 308 samples responded before the opening in the 2007 survey [16].

Table 3 displays the number of samples, sorted in terms of estimated noise levels and the existence of conventional railways in the residential area. The Hokuriku Shinkansen railway currently runs from Tokyo to Kanazawa Station. Therefore, the area to the west of the Kanazawa Station only has conventional railways, and the number of respondents in this area was 95. In areas with and without conventional railways, the housing structure was more than 90% wooden. Ninety-two Hokuriku Shinkansen railway trains passed per day in these areas, and the maximum speed in the areas with and without conventional railways were in the ranges of 150 km/h to 208 km/h and 195 km/h to 250 km/h, respectively.

### 3.2. Exposure–Response Relationships for Hokuriku Shinkansen Railway

**Figure 3:**
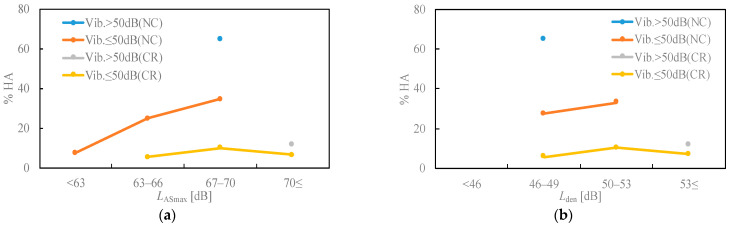
Relationships between noise exposure level and HA. (**a**) *L*_ASmax_; (**b**) *L*_den_.

Figure 3 displays the relationship between the noise exposure level and noise annoyance. Noise annoyance near a conventional railway area evoked a nearly constant response to increases in *L*_ASmax_ and *L*_den_, and the noise annoyance was high in areas where the vibration level was high. The result that the noise annoyance was greater in areas where the ground vibration level was high is similar to those in the literature [9,10,23]. Furthermore, the response of the area where the conventional railway does not run parallel to the Shinkansen railway (NC) in the range of 63–70 dB (*L*_ASmax_) and 46–53 dB (*L*_den_) was much higher than that of the conventional railway area.

**Figure 4:**
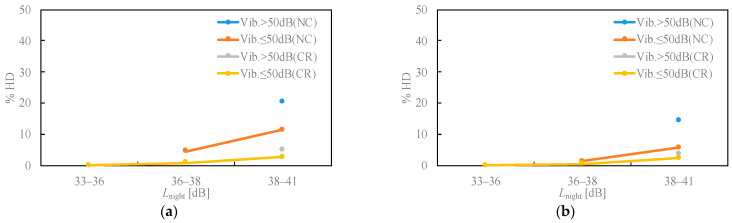
Relationships between noise exposure level and self-reported sleep disturbances. (**a**) Falling asleep; (**b**) awakening.

Figure 4 displays the relationship between the falling asleep disturbance and awakening. The falling asleep disturbance in the NC area was high, in the range of 38–41 dB *L*_night_. In addition, the response to a vibration level of over 50 dB was even higher. Regarding awakening in the same noise level range, the response tended to be smaller than that of the falling asleep disturbance. The fact that the Hokuriku Shinkansen railway does not operate between 24:00 and 6:00 may have slightly influenced the awakening results. The operation of conventional railways is limited to freight trains after 24:00, and normal trains resume at approximately 5:00.

**Figure 5:**
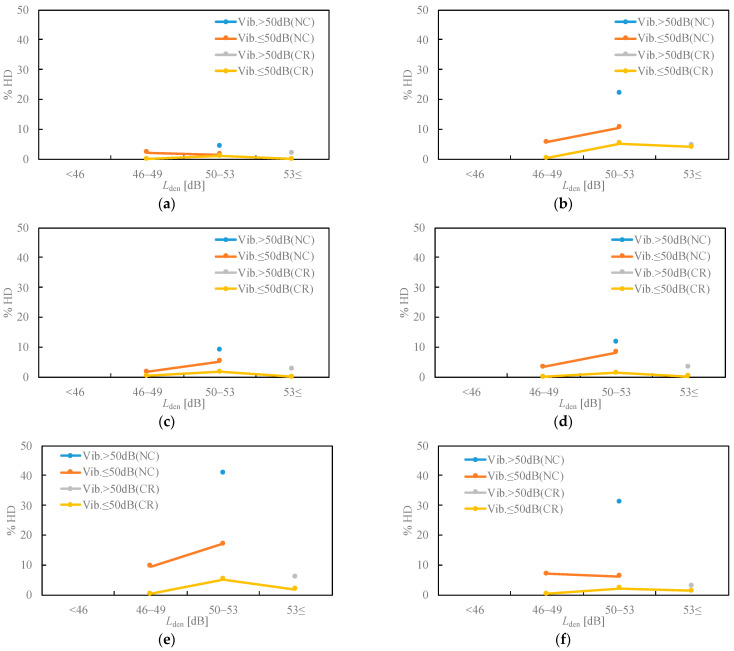
Relationships between noise exposure level and activity disturbance. (**a**) Conversation; (**b**) TV/radio listening; (**c**) reading; (**d**) thinking; (**e**) window open; (**f**) rattling.

Figure 5 displays the results of activity disturbances. The conversation and reading disturbances were low, within the range of 44–55 dB *L*_den_. For television/radio listening disturbance, the responses in NC and high-vibration-level areas were slightly higher, and 22% were highly disturbed in the range of 50–53 dB *L*_den_. The thinking disturbance was low in the conventional railway areas and did not depend on *L*_den_, but the responses in the NC area were slightly higher than those in the conventional area. The discomfort that the windows cannot be opened due to noise and the rattling in the NC area was higher than that in the conventional railway area. In particular, the response rates for rattling were in the range of 50–53 dB *L*_den_ in the NC area, and the rate of large vibrations in residential areas was high.

Table 4, Table 5, Table 6 and Table 7 present the multiple logistic regression analysis of noise annoyance and self-reported sleep disturbances. In all analyses, the interaction between noise level and the vibration level, and the interaction between noise level and the presence of conventional railway, gender, age, and family size, were not significant and were excluded from the analysis results. The effects of both *L*_ASmax_ and *L*_den_ on noise annoyance were significant at the 5% level, as illustrated in Table 4 and Table 5. The vibration level was not significant in both noise annoyance models in the area with a conventional railway. The odds ratio of noise annoyance in the area without a conventional railway was significantly higher than that with one. The vibration levels in the area without a conventional railway were also significant in both noise annoyance models. The area under the curve (AUC) values in Table 4 and Table 5 were all greater than 0.7. The HSD was not affected by *L*_night_, but it was significantly affected by the vibration level and the existence of a conventional railway (Table 6). The existence of a conventional railway in the vicinity also had a significant effect on the community response. The dependent variable in Table 7 is the frequency of awakening during sleep. In this analysis, those who selected four (2–3 times a week) or more were treated as one. *L*_night_, the vibration level, and the existence of a conventional railway exhibited a significant effect on the awakening frequency. It was also shown that noise annoyance and the sleep disturbance of people who are sensitive to noise were also significantly higher.

### 3.3. Changes in the Evaluation to Living Environmental Factors: Housing Satisfaction, Preference for the Residential Area and Quietness around the House

This section presents the results of whether noise and vibration caused by the Hokuriku Shinkansen railway affected housing satisfaction, residential area preference, and quietness around the house, according to the survey year. There were 308 residents who responded to both the 2007 and 2016 surveys. These three items were asked in both surveys, and each was evaluated on a five-point scale, although the terms of the scales were different (Table 1). The items were divided into three conditions according to the distance between the Shinkansen rail track and each house (within 80 m and further than 80 m) and the control area. The range of distance from the Shinkansen rail track to the houses in these data was from 11 m to 139 m, and the median distance was 78 m. Table 8 displays the tabulation and Fisher’s exact test for housing satisfaction. The housing dissatisfaction in the areas over 80 m away was slightly lower in 2016 than in 2007, but this was not significantly different from the three conditions in the survey year. Table 9 displays the tabulation and Fisher’s exact test for the preference for the residential area. The evaluations on the dislike side were all lower than 10%, and there was no difference in the survey year. Table 10 displays the tabulation and Fisher’s exact test for the quietness around the house. The evaluation of the bad side in the area near to the rail track was higher than in the far area, but there was no difference between the survey years in any conditions. We also analyzed the soundproof levels, the natural environment, the view from own house, and the landscape as factors that may affect the evaluations of the living environment. However, there was also no significant differences in these items over time.

## 4. Discussion

The study results exhibited a relationship between *L*_den_ and noise annoyance (Figure 3). Regarding the conditions of residential areas running parallel to a conventional railway line, the %HA of 46–53 dB in our results was almost the same as the results (survey II) after the opening at the Kyushu Shinkansen [5]. On the other hand, the% HA of the Nagano Shinkansen survey [24] at the same level was quite low, and less than 1%. The Nagano Shinkansen survey was conducted about 15 years after its opening in 1998, and the length of the elapsed year may have led to a decline in% HA.

The result (Figure 3, Figure 4 and Figure 5) that the existence of a conventional railway affects noise annoyance suggests the possibility of a change effect, because the survey area where there was no conventional railway was quiet. Brown and van Kamp [25] reviewed studies that investigated responses to changes in transportation noise and found that transparent information and communication concerning noise changes can positively affect community attitudes and expectations. In addition, some community responses, such as noise annoyance, were lower in areas where conventional lines exist than in areas where there were no conventional railways; it is also possible that accustomed to noise and vibration caused by the railway is affecting annoyance and daily activity disturbances. Continuous surveys are required to verify the drastic change effect and habituation to the acoustic environment.

Our study confirmed that noise annoyance was affected by the vibration level and existence of a conventional railway near the residential area. Noise annoyance was high in areas where the vibration level was high, and the characteristics are particularly notable in areas where a conventional railway does not run parallel to the homes. When considering guideline values for the Shinkansen railway noise, it may be necessary to consider the degree of ground vibration level and the existence of conventional lines in the area. This finding is apparent from the multiple logistic regression analysis results, and the odds ratio for the residents in areas where there are no conventional lines was approximately five times higher than that in areas with a conventional line. The findings concerning the vibration effect are the same as those of Yano et al. [4] and Yokoshima et al. [7]. Yano et al. [4] demonstrated that the general noise annoyance was higher along the Shinkansen railway than along the conventional railway for the same noise level. They also showed that the vibration annoyance was high, suggesting the effect of vibration on noise annoyance. Yokoshima et al. [7] also demonstrated that the noise annoyance differs depending on the vibration level on the ground, and the noise annoyance was low with low levels of vibration. Sleep disturbance was also affected by both the vibration exposure and the presence of a conventional railway. When constructing a new Shinkansen line in a residential area that does not have sound and vibrations from a conventional railway, taking vibration-proof measures and providing explanations to the residents regarding the noise and vibrations caused by the Shinkansen railway may effectively reduce noise annoyance and sleep disturbance.

Zhang et al. [26] reported that satisfaction with housing and living environment was related to noise annoyance, sleep disturbance, and certain activity disturbances in a logistic regression analysis. Izumi et al. [27] illustrated that the impression of a residential area directly affects road traffic noise annoyance. Morihara et al. [28], through structural equation modeling, also demonstrated that annoyance due to railway noise was affected by the living environment evaluation. Schreckenberg et al. [29] examined the factors related to quietness using linear multiple regression models and illustrated that aircraft noise (*L*_Aeq,16 h_, not road traffic noise), noise sensitivity, and mental health significantly affected the perceived quietness. Therefore, we considered that there was some relationship between noise annoyance and housing satisfaction, preference for the residential area, and quietness around the house, and compared the 2007 survey data, before the opening of the Hokuriku Shinkansen, with the 2016 survey data, after the opening. The conditions for the comparison were three areas: one was a control area where the Shinkansen did not operate and the others were divided into two areas according to the distance from the Shinkansen rail track, considering the effects of noise and vibration. As a result, the evaluation of these three items dealt with in this study did not worsen with the opening of the Hokuriku Shinkansen railway. In this survey area, conventional railways run in parallel with the Shinkansen railway, and the speed of the Shinkansen railway was slow; therefore, it was possible that they did not contribute to the evaluation of the negative effects of these three items. Only 34 out of 125 houses had a 2016 Shinkansen *L*_Aeq,24 h_ of at least 3 dB higher than the 2007 estimated *L*_Aeq,24 h_ of a conventional railway. In other words, the noise exposure level in this survey area did not increase as much due to the opening of the Hokuriku Shinkansen. It may be necessary to compare before-and-after surveys in areas where there are no conventional railways, and the speed of the Shinkansen is fast.

## 5. Conclusions

This study investigated whether noise annoyance and activity disturbances owing to the Shinkansen railway, including self-reported sleep disturbance, were affected by noise and vibration exposure levels and the existence of conventional railways.

Regarding noise annoyance, a high percentage of HA respondents live in high-vibration areas and areas without conventional railways. Specifically, the annoyance of residents in areas where conventional railways exist was not different from the results of previous studies [5], but the annoyance level of residents in areas where there was no conventional railway was about 20% higher, and in areas where vibration due to the Shinkansen exceeds 50 dB, it was even higher, in the range of 46–49 dB *L*_den_. It was shown that, when establishing guidelines for high-speed railways, it may be necessary to consider the existence of conventional railways in the residential area and the effects of vibrations due to the Shinkansen railway. However, because the results of this study were based on social survey data conducted one year after the opening of the Shinkansen railway, the differences in the responses of communities with and without conventional railways may be due to this drastic change. Although not to the same degree as that for noise annoyance, similar trends were observed for activity disturbances (e.g., sleep, television/radio listening, thinking, window opening, and rattling disturbances). These survey data show that, regarding the evaluation of the negative effects on housing satisfaction, the preference for the residential area and quietness around the house did not worsen with the opening of the Hokuriku Shinkansen railway.

In Japan, prior explanations for and counter measures against noise were taken in the areas along the Hokuriku Shinkansen lines before the opening, but in the area where the conventional railway does not run parallel to the Shinkansen railway, the noise annoyance was high. The 2007 survey did not conduct a before survey in areas where conventional railways do not run in parallel; therefore, the results of this study before and after the opening are limited to the area where the conventional railway exists. In the next step, we will conduct socio-acoustic surveys before and after the opening, looking at effects of noise and vibration in areas where conventional railways are not running in parallel. In addition, hierarchical causal models of noise annoyance and housing satisfaction should be investigated to understand the extent to which noise and vibrations affect community responses.

## Figures and Tables

**Table 1:** Question words and evaluation scales.

Q1. How satisfied are you with your current home?
“1 Extremely satisfaction, 2 Satisfaction, 3 Normal, 4 Dissatisfaction, 5 Extremely dissatisfaction”
Q2. How much do you like the area where you live?
“1 Like very much, 2 Like, 3 Neither like nor dislike, 4 Dislike, 5 Dislike very much”
Q3. Please evaluate your living area according to the following items. 4 Quietness around own house
“1 Extremely good, 2 Good, 3 Neutral, 4 Bad, 5 Extremely bad”

**Table 2:** Demographic attributes.

Age	Gender (*n* (%))			Family Size	(*n* (%))	Sensitivity WNS-6B	(*n* (%))
	Male	Female	Total				
10s	4 (0.4)	6 (0.6)	10 (1.0)	one	116 (11.5)	0	42 (4.2)
20s	14 (1.4)	12 (1.2)	26 (2.6)	two	341 (33.8)	1	44(4.4)
30s	21 (2.1)	19 (1.9)	40 (3.9)	three	240 (23.8)	2	116(11.6)
40s	60 (5.9)	64 (6.3)	124 (12.2)	four	180 (17.8)	3	150(15.1)
50s	100 (9.9)	91 (9.0)	191 (18.8)	five	75 (7.4)	4	209(21.0)
60s	185 (18.2)	157 (15.5)	342 (33.7)	six	36 (3.6)	5	242(24.3)
70s or more	187 (18.4)	94 (9.3)	281 (27.7)	seven	16 (1.6)	6	193(19.4)
				eight	5 (0.5)		
				nine	1 (0.1)		
Total	571 (56.3)	443 (43.7)	1014 (100)	Total	1010 (100)	Total	996 (100)

**Table 3:** Sample sizes divided by noise level.

*L*_ASmax_ [dB]	CR ^1^		Total	*L*_den_ [dB]	CR		Total	*L*_nihgt_ [dB]	CR		Total
	With	Without			With	Without			With	Without	
West ^2^	95	-	95	West	95	-	95	West	95	-	95
<63	7	30	37	<46	1	19	20	<36	105	15	120
63–66	138	128	266	46–49	122	191	313	36–38	344	61	405
67–70	254	149	403	50–53	225	105	330	38<	146	256	402
70<	196	25	221	53<	247	17	264				
Total	690	332	1022	Total	690	332	1022	Total	690	332	1022

^1^ CR: Conventional Railway, ^2^ West: this is west of Kanazawa Station, the control area.

**Table 4:** Multiple logistic regression analysis of high noise annoyance by *L*_ASmax_, *L*_Vmax_ and existence of conventional railway (AUC = 0.754).

Items	Category	Estimate	Standard Error	*p*-Value	Odds Ratio	95% Confidence Interval	
						Lower	Upper
Intercept		−15.404	3.155	0.000			
*L*_ASmax_		0.186	0.046	0.000	1.204	1.101	1.317
*L*_Vmax_ & existence of Conventional railway	*L*_Vmax_ ≤ 50&CR				1		
	*L*_Vmax_ > 50&CR	−0.526	0.451	0.243	0.591	0.244	1.429
	*L*_Vmax_ ≤ 50&NC	1.595	0.223	0.000	4.929	3.186	7.625
	*L*_Vmax_ > 50&NC	2.586	0.383	0.000	13.281	6.264	28.160
Noise sensitivity	Not sensitive				1		
	Sensitive	0.795	0.195	0.000	2.214	1.510	3.246

**Table 5:** Multiple logistic regression analysis of high noise annoyance by *L*_den_, *L*_Vmax_ and existence of conventional railway (AUC = 0.747).

Items	Category	Estimate	Standard Error	*p*-Value	Odds Ratio	95% Confidence Interval	
						Lower	Upper
Intercept		−11.504	2.600	0.000			
*L*_den_		0.170	0.049	0.001	1.185	1.075	1.306
*L*_Vmax_ & existence of Conventional railway	*L*_Vmax_ ≤ 50&CR				1		
	*L*_Vmax_ > 50&CR	−0.313	0.442	0.479	0.731	0.308	1.738
	*L*_Vmax_ ≤ 50&NC	1.770	0.253	0.000	5.868	3.576	9.628
	*L*_Vmax_ > 50&NC	2.950	0.390	0.000	19.115	8.895	41.077
Noise sensitivity	Not sensitive				1		
	Sensitive	0.810	0.019	0.000	2.247	1.535	3.290

**Table 6:** Multiple logistic regression analysis of high sleep disturbance by *L*_night_, *L*_Vmax_ and existence of conventional railway (AUC = 0.863).

Items	Category	Estimate	Standard Error	*p*-Value	Odds Ratio	95% Confidence Interval	
						Lower	Upper
Intercept		−13.330	4.292	0.002			
*L*_night_		0.207	0.114	0.069	1.230	0.984	1.536
*L*_Vmax_ & existence of Conventional railway	*L*_Vmax_ ≤ 50&CR				1		
	*L*_Vmax_ > 50&CR	1.245	0.755	0.099	3.471	0.791	15.244
	*L*_Vmax_ ≤ 50&NC	1.830	0.561	0.001	6.232	2.077	18.698
	*L*_Vmax_ > 50&NC	2.568	0.723	0.000	13.044	3.161	56.826
Noise sensitivity	Not sensitive				1		
	Sensitive	1.698	0.406	0.000	5.465	2.465	12.116

**Table 7:** Multiple logistic regression analysis of the frequency of the awakening by *L*_night_, *L*_Vmax_ and existence of conventional railway (AUC = 0.835).

Items	Category	Estimate	Standard Error	*p*-Value	Odds Ratio	95% Confidence Interval	
						Lower	Upper
Intercept		−11.716	3.203	0.000			
*L*_night_		0.0197	0.085	0.020	1.218	1.031	1.439
*L*_Vmax_ & existence of Conventional railway	*L*_Vmax_ ≤ 50&CR				1		
	*L*_Vmax_ > 50&CR	1.144	0.471	0.015	3.140	1.248	7.901
	*L*_Vmax_ ≤ 50&NC	1.422	0.357	0.000	4.146	2.058	8.353
	*L*_Vmax_ > 50&NC	1.948	0.539	0.000	7.012	2.439	20.160
Noise sensitivity	Not sensitive				1		
	Sensitive	1.629	0.279	0.000	5.100	2.953	8.808

**Table 8:** Cross-tabulation and Fisher’s exact test for the housing satisfaction.

Conditions	Category	Dissatisfaction N (%)	The Others N (%)	Fisher’s Exact Test (s-Sided)
*Distance* ≤ 80 m	2007	18 (13)	116 (87)	0.711
	2016	15 (11)	118 (89)	
*Distance* > 80 m	2007	12 (11)	97 (89)	0.218
	2016	6 (6)	103 (94)	
*Control area*	2007	9 (15)	51 (85)	0.602
	2016	7 (11)	54 (89)	

**Table 9:** Cross-tabulation and Fisher’s exact test for the preference for the residential area.

Conditions	Category	Dislike N (%)	The Others N (%)	Fisher’s Exact Test (s-Sided)
*Distance* ≤ 80 m	2007	9 (7)	124 (93)	0.168
	2016	4 (3)	130 (97)	
*Distance* > 80 m	2007	4 (4)	104 (96)	0.720
	2016	3 (3)	107 (97)	
*Control area*	2007	3 (5)	57 (95)	0.619
	2016	1 (2)	59 (98)	

**Table 10:** Cross-tabulation and Fisher’s exact test for the quietness.

Conditions	Category	Bad N (%)	The Others N (%)	Fisher’s Exact Test (s-Sided)
*Distance* ≤ 80 m	2007	42 (31)	92 (69)	0.894
	2016	39 (30)	91 (70)	
*Distance* > 80 m	2007	13 (12)	97 (88)	0.840
	2016	15 (13)	97 (87)	
*Control area*	2007	15 (25)	45 (75)	1.000
	2016	15 (25)	46 (75)	

## Data Availability

Data sharing not applicable.
